# Computational Protocol to Evaluate Electron–Phonon Interactions Within Density Matrix Perturbation Theory

**DOI:** 10.1021/acs.jctc.2c00579

**Published:** 2022-09-20

**Authors:** Han Yang, Marco Govoni, Arpan Kundu, Giulia Galli

**Affiliations:** † Department of Chemistry, University of Chicago, Chicago, Illinois 60637, United States; ‡ Pritzker School of Molecular Engineering, The University of Chicago, Chicago, Illinois 60637, United States; § Materials Science Division and Center for Molecular Engineering, Argonne National Laboratory, Lemont, Illinois 60439, United States

## Abstract

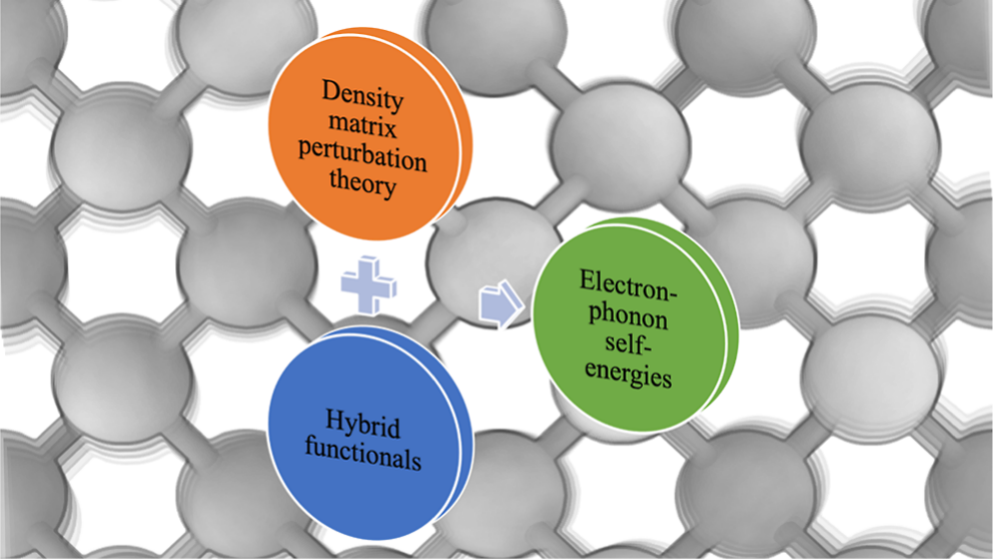

We present a computational protocol, based on density matrix perturbation theory, to obtain non-adiabatic, frequency-dependent electron–phonon self-energies for molecules and solids. Our approach enables the evaluation of electron–phonon interaction using hybrid functionals, for spin-polarized systems, and the computational overhead to include dynamical and non-adiabatic terms in the evaluation of electron–phonon self-energies is negligible. We discuss results for molecules, as well as pristine and defective solids.

## 1 Introduction

The study of electron–phonon interaction in solids can be traced back to the early days of quantum mechanics,^1^ and it has been instrumental in explaining fundamental properties of solids, including conventional superconductivity.^2^ However, it was not until recent years that electron–phonon interaction was computed from first-principles,^3−7^ leading to non-phenomenological predictions of transport properties of solids^8^ and of electron–phonon renormalizations of band structures.^7,9−13^ Although early studies relied on semi-empirical models^14−16^ to study electron–phonon interaction, modern investigations typically employ the frozen phonon (FPH) approach,^7,17^ density functional perturbation theory (DFPT),^4,5,10,11,18^ or molecular dynamics (MD) simulations,^12,19,20^ with electron–electron and electron–ion interactions described at the level of density functional theory (DFT).^21^

In two recent papers,^10,11^ we combined first-principles calculations of electron–electron and electron–phonon self-energies in molecules and solids, within the framework of DFPT. We developed an approach that enables the evaluation of electron–phonon coupling at the *G*_0_*W*_0_ level of theory^22−25^ for systems with hundreds of atoms and the inclusion of non-adiabatic and temperature effects at no additional computational cost. Recent developments have also extended the DFPT method to study electron–phonon interactions using DFT + U^26^ to obtain an improved description of systems with strong electronic correlation. We also computed^12^ electron–phonon renormalizations of energy gaps by using the path-integral MD (PIMD) methods to investigate anharmonic effects in crystalline and amorphous solids. The DFPT-, FPH-, and MD-based methods are addressing different regimes and different problems; the use of DFPT and FPH is appropriate for systems whose atomic constituents all vibrate close to their equilibrium positions, although anharmonic effects have been included in some FPH calculations.^7^ The assumption of close to equilibrium vibrations is however not required when applying PIMD, which thus has a wider applicability; for example, it can be used to study amorphous materials and molecules and solids exhibiting prominent anharmonic effects, for example, molecular crystals^27,28^ and several perovskites.^29^ However, the calculation of electron–phonon renormalizations using FPH- and MD-based methods is carried out within the Allen–Heine–Cardona (AHC)^30,31^ formalism, which neglects dynamical and non-adiabatic terms of the electron–phonon self-energies. These effects have been shown to be essential to describe electron–phonon interactions in numerous polar materials,^32,33^ for example, SiC. Perturbation-based methods, on the other hand, can accurately compute electron–phonon self-energies within and beyond the AHC formalism, thus including non-adiabatic and/or frequency-dependent effects into the self-energy. Another benefit of DFPT-based methods is the ability to explicitly evaluate the electron–phonon coupling matrices, which are useful quantities, for example, in the study of mobilities^34−40^ and polaron formation.^41−46^

Here, we generalize the perturbation-based approach of refs (10) and (11) to enable efficient calculations of electron–phonon interaction with hybrid functionals, by using density matrix perturbation theory (DMPT)^47,48^ to compute phonons and electron–phonon coupling matrices. Our implementation takes advantage of the Lanczos algorithm,^49^ which enables the calculations of electron–phonon self-energies beyond the AHC approximation, at no extra cost.

DMPT has been used in the literature to compute excitation energies and absorption spectra in molecules and solids in conjunction with time-dependent DFT (TDDFT)^50,51^ and to solve the Bethe–Salpeter equation (BSE).^48,52−56^ In the latter case, DMPT has been applied to obtain the variation of single-particle wavefunctions due to the perturbation of an electric field. However, DMPT is a general formalism that can be used to compute the response of a system to perturbations of any form, including perturbations caused by atomic displacements.

In this paper, we first derive a formalism for phonon calculations within DMPT, starting from the quantum Liouville equation in Section 2; we then verify our results by comparing them with those of FPH and PIMD calculations in Section 3. We then present calculations of electron–phonon interactions in small molecules (Section 4) and pristine (Section 5) and defective diamonds (Section 6) using hybrid functionals, and we conclude the paper in Section 7 with a summary of our findings.

## 2 Methodology

Using Hartree atomic units (*ℏ* = *e* = *m*_e_ = 1), we describe the electronic structure of a solid or molecule within Kohn–Sham (KS) DFT, and we consider the quantum Liouville’s equation to describe perturbations acting on the system
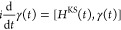
1where [·, ·] denotes a commutator and *H*^KS^(*t*) is the KS Hamiltonian
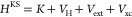
2with *K* as the kinetic operator, *V*_H_ as the Hartree potential, *V*_ext_ as the external potential, and *V*_xc_ as the exchange–correlation potential. The KS Hamiltonian does not depend explicitly on time and depends implicitly on time through the time-dependent density matrix γ that can be written in terms of KS single-particle orbitals ψ_nσ_
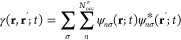
3where σ is the spin polarization, *n* is the band index, and *N*_occ_^σ^ is the number of occupied bands in the spin channel σ. Below we present calculations performed by sampling the Brillouin zone with only the Γ point and hence omit labeling of eigenstates with **k**-points.

Given a time-dependent perturbation ∂*V*_ext_(*t*) acting on the Hamiltonian, the first-order change in the density matrix ∂γ(*t*) satisfies the following equation
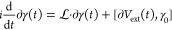
4where  is the Liouville super-operator
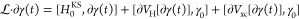
5

Here, we use the notation ∂ to represent a change in potentials (∂*V*), wavefunctions ∂ψ, charge densities ∂ρ(**r**), and density matrices ∂γ(**r**, **r**′); γ_0_ and *H*_0_^KS^ are the density matrix and the KS Hamiltonian of the unperturbed system, respectively.

Taking the Fourier transform of eq 4, we rewrite it in the frequency domain
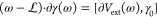
6

In phonon calculations, we adopt the Born-Oppenheimer approximation,^57^ and no retardation effects are included. Hence, we only need to solve eq 6 at ω = 0
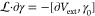
7

The equation above can be cast in the following form
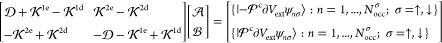
8where  is the projection operator onto the virtual bands; , , , and , defined below in eqs 12 and 13 and 15–18, are related to the variation of exchange–correlation potential *V*_xc_; the elements of the arrays  and  are variations of wavefunctions; the variation of the density matrix in terms of wavefunction variation is
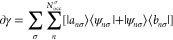
9

In phonon calculations, the external perturbation is static ∂*V*_ext_(ω = 0), and eq 8 can be further simplified since *a*_*n*σ_ = *b*_*n*σ_ = ∂ψ_*n*σ_ for static perturbations, and eq 8 becomes
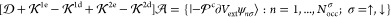
10

Equation 10 is a generalized Sternheimer equation,^58^ where the operators on the left-hand side are defined below.
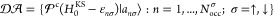
11

When using LDA/GGA functionals, the  and  operators are
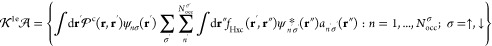
12
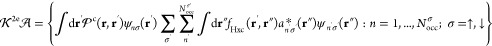
13where *f*_Hxc_ = *v*_c_ + *f*_xc_ is the sum of the bare Coulomb potential *v*_*c*_ and the exchange–correlation kernel
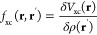
14with ρ(**r**) being the electron density.

When using hybrid functionals, the operators are
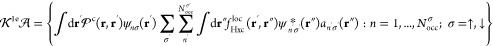
15
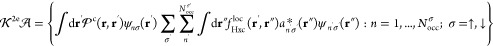
16
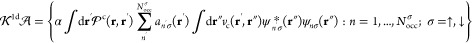
17
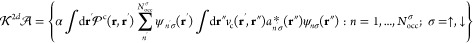
18where *f*_Hxc_^loc^ = *v*_c_ + *f*_xc_^loc^ is the sum of the bare Coulomb potential and the local part of the exchange–correlation kernel
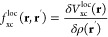
19and the parameter α is the fraction of the Hartree–Fock exchange included in the definition of the hybrid functional. Note that the  and  operators are zero for LDA/GGA functionals.

Once we have the solutions *a*_*n*σ_ of the Liouville equation (eq 8 or eq 10), that is, the change in wavefunction ∂ψ_*n*σ_, we can compute the change in the density matrix with eq 9; the change in density is then given using the following expression
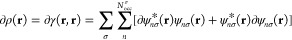
20and force constants are obtained as follows
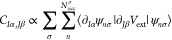
21

By diagonalizing the dynamical matrix
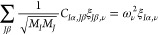
22where *M*_*I*_ and *M*_*J*_ are atomic masses, we obtain the frequency ω_ν_ of mode ν and its polarization ξ_*I*α,ν_.

To compute the electron–phonon coupling matrices in the Cartesian basis
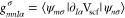
23or in the phonon-mode basis
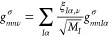
24where ξ_*I*α,ν_ is the νth vibrational mode, we need to evaluate the change in the self-consistent (scf) potential ∂*V*_scf_. The scf potential is given by the sum of the Hartree potential *V*_H_, the local part of the exchange–correlation potential *V*_xc_^loc^, and the non-local Hartree-Fock exchange *V*_xc_^nl^. Thus, the change in the scf potential ∂*V*_scf_|ψ_*n*σ_⟩ is the sum of the following three terms
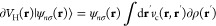
25
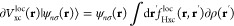
26and
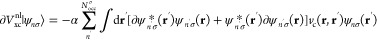
27

The Fan–Migdal and Debye–Waller self-energies can then be computed as
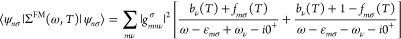
28
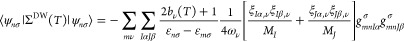
29where *b*_ν_ is the occupation number of the frequency ω_ν_ obeying the Bose–Einstein distribution and *f*_*m*σ_ is the occupation number of the KS eigenvalues ε_*m*σ_ obeying the Fermi-Dirac distribution. The Debye–Waller self-energy is derived within the rigid-ion approximation (RIA),^30,59,60^ which approximates second-order electron–phonon coupling matrices with first-order ones.

Using the frequency-dependent Fan–Migdal self-energy, the renormalized energy levels can be evaluated self-consistently
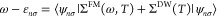
30with initial guess ω_0_ = ε_*n*σ_ and using the Lanczos^49^ algorithm to evaluate the frequency-dependent Fan–Migdal self-energy (for a detailed derivation, see refs (10) and (11)). Calculations using full frequency-dependent self-energies with DFPT have been recently reported in the literature.^10,32,43^

We refer to the FM self-energy in eq 28 as the non-adiabatic fully frequency-dependent (NA-FF) self-energy. If the frequency dependence is considered within the adiabatic approximation, the self-energy is
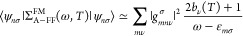
31

We refer to eq 31 as the adiabatic fully frequency-dependent (A-FF) self-energy.

In our formulation the evaluation of self-energies can be carried out simultaneously at multiple frequencies using the Lanczos algorithm; however, we introduce below approximations leading to frequency-independent self-energies for comparison with results present in the literature, obtained, for example, with the AHC formalism.^30,31^ In particular, we evaluate the FM self-energy by applying the so-called on-the-mass-shell (OMS) approximation, that is, by setting ω = ε_*n*σ_ in the expressions of the A-FF and NA-FF self-energies. In the former case, we obtain the adiabatic AHC (A-AHC)^30,31^ approximation, and in the latter case, we obtain the non-adiabatic AHC (NA-AHC) approximation
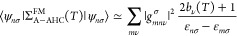
32
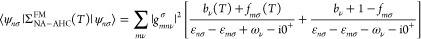
33

We summarize the various levels of approximations applied to evaluate the FM self-energy in Table 1; the corresponding DW self-energies are the same for all levels of approximation. Thus, we also use the acronyms A-AHC, NA-AHC, A-FF, and NA-FF to denote the level of theory adopted for the total self-energy (FM + DW) and for the electron–phonon renormalization of fundamental gaps.

**Table 1:** List of Theoretical Approximations Used in This Paper to Compute the Fan–Migdal Self-Energy, Where We Specify Whether the On-The-Mass-Shell (OMS) and the Adiabatic Approximation are Adopted (√) or Not Adopted (×)

Level of Theory	OMS	Adiabatic	Equation
(A-)AHC^a^	√	√	32
NA-AHC	√	×	33
A-FF	×	√	31
NA-FF	×	×	28

a In the main text, we use AHC and A-AHC interchangeably.

## 3 Verification

To verify the implementation of the method described above in the WEST^24^ package, we first computed the phonon frequencies of selected solids (diamond, silicon, and silicon carbide) and the vibrational modes of selected molecules (H_2_, N_2_, H_2_O, and CO_2_) and compared our results with those of the FPH approach. The displacements used for FPH calculations are 0.001 Å for all molecules and solids. In Tables 2 and 3, we summarize our results obtained at the PBE0^61^ level of theory and obtained by solving either the Liouville equation or by using the FPH approach. The lattice constants used for diamond, silicon, and silicon carbide are 3.635, 5.464, and 4.372 Å, respectively, and the cell used for molecules is a cube of edge 10.583 Å. For verification purposes, we only computed the phonon modes at the Γ point in the Brillouin zone of the solids. We used an energy cutoff of 60 Ry for the solids and 50 Ry for the molecules and the SG15^62^ ONCV^63^ pseudopotentials for all solids and molecules.

**Table 2:** Comparison of Selected Phonon Frequencies [cm^–1^] in Diamond, Silicon, and Silicon Carbide Computed in a Primitive Cell With the PBE0 Functional by Solving the Liouville’s Equation or by Using the FPH Approach

Solid	Liouville	FPH	Absolute Difference
Diamond	2136.21	2131.48	4.73
Silicon	737.47	737.28	0.19
silicon carbide	612.77	612.70	0.07

**Table 3:** Comparison of the Vibrational Modes [cm^–1^] of Selected Molecules Obtained With the PBE0 Functional and Computed by Solving the Liouville’s Equation or by Using the FPH Approach^a^

Molecule	Symmetry	Liouville	FPH	Absolute Difference
H_2_	a_1_	4421.62	4421.48	0.14
N_2_	a_1_	2480.36	2480.36	0.00
H_2_O	a_1_	1652.79	1658.76	5.97
H_2_O	b_2_	3921.28	3936.57	15.29
H_2_O	a_1_	4033.68	4048.58	11.90
CO_2_	e_1u_	698.15	698.12	0.03
CO_2_	a_1g_	1375.10	1375.18	0.08
CO_2_	a_1u_	2419.08	2419.23	0.15

a The symmetry of the mode is given in the second column.

Table 2 shows that the absolute difference in the phonon frequencies computed with the FPH approach and the method implemented here is small for silicon and silicon carbide, 0.19 and 0.07 cm^–1^, respectively. The corresponding difference for diamond is larger but still acceptable being below 5 cm^–1^. In Table 3, we compare the vibrational frequencies of H_2_, N_2_, H_2_O, and CO_2_ molecules computed by solving the Liouville equation and applying the frozen-phonon (FPH) approach. We found again that the differences are small for H_2_, N_2_, and CO_2_ (below 1 cm^–1^), albeit slightly larger for H_2_O. The largest difference is found in the case of H_2_O (15.29 cm^–1^), and this is most likely due to the numerical inaccuracy of the FPH approach.

To verify our calculations of electron–phonon interactions, we carried out a detailed study of the renormalization of the HOMO–LUMO gaps (*E*_*g*_) of the CO_2_, Si_2_H_6_, HCN, HF, and N_2_ molecules, with the results for CO_2_ being summarized in Table 4 and the rest in Table 5.

**Table 4:** Electron–Phonon Renormalization Energies [meV] of HOMO and LUMO Energy Levels and the HOMO–LUMO Gap in the CO_2_ Molecule, Computed by Solving Liouville’s Equation, Using DFPT, the FPH Approach, and the PIMD Method^a^

Method	Functional	HOMO Renorm.	LUMO Renorm.	Gap Renorm.
Liouville	LDA	64	–453	–517
DFPT	LDA	64	–453	–517
Liouville	PBE	65	–350	–415
DFPT	PBE	65	–350	–415
FPH	PBE	53	–325	–378
Liouville	PBE0	68	–69	–137
FPH	PBE0	55	–77	–132
PIMD	PBE0	59	–103	–162
Liouville	B3LYP	67	–107	–174
FPH	B3LYP	54	–89	–143
PIMD	B3LYP	58	–112	–170
				
Reference (69)	LDA			–680.7
	PBE + TS			–716.2
	B3LYP			–4091.6

a We compare results obtained with different functionals, LDA, PBE, PBE0, and the B3LYP functionals, and include results obtained in ref (69).

**Table 5:** Electron–Phonon Renormalization Energies [meV] of the Energy Gap in Si_2_H_6_, HCN, HF, and N_2_ Molecules Computed by Solving Liouville’s Equation and Using the Frozon Phonon (FPH) Approach at the B3LYP Level of Theory

Molecule	Liouville	FPH	Ref (69)
Si_2_H_6_	–117	–139	–1872.3
HCN	–19	–14	–171.4
HF	–18	–25	–29.9
N_2_	8	–6	8.7

Table 4 summarizes the renormalizations to the *E*_g_ of the CO_2_ molecule obtained within the A-AHC formalism and using DFPT, FPH, and PIMD^12^ at the LDA,^64^ PBE,^65^ PBE0,^61^ and B3LYP^66−68^ levels of theory, respectively. With the LDA and PBE/GGA functionals, the solution of the Liouville equation yields the same results as the method proposed in refs (10) and (11), as expected. When solving the Liouville equation with the DFPT method, the RIA is adopted; however, the latter approximation is not used in the FPH approach, leading to a slight difference between the FPH and Liouville results. In addition, we carried out calculations with the hybrid functionals, PBE0 and B3LYP, and compared our results with those of the FPH and PIMD approaches.^12^ The PIMD approach circumvents the RIA and also includes ionic anharmonic effects. Since the RIA is adopted and anharmonicity is not included in the Liouville approach, differences on the order of ∼30 meV, relative to PIMD, are considered as acceptable. We note that the computed renormalizations of the gap of CO_2_ reported in the literature,^69^ –680.7 and −716.2 meV with LDA^64^ and PBE + TS^70^ functionals, respectively, are significantly different from those obtained here. We also note that ref (69) reports the result at the B3LYP level of theory, – 4091.6 meV, which is one order of magnitude larger than the corresponding LDA and PBE + TS results, hence calling into question the numerical accuracy of the data. Such significant differences between our and the results of ref (69) probably stems from the different choices of basis functions, localized basis functions in ref (69) and plane waves in this work.

In addition to CO_2_, we also computed the energy gap renormalizations of Si_2_H_6_, HCN, HF, and N_2_ molecules with the B3LYP functional; these are shown in Table 5. For Si_2_H_6_ and HCN, the results computed with Liouville’s equation and the FPH approach agree well, with small differences of 22 and 5 meV, respectively. The renormalization of HF is about −20 meV with both the Liouville and FPH approaches, consistent with the result −29.9 meV reported in the literature. The Liouville and FPH methods both predict the renormalization of N_2_ to be close to zero, in agreement with ref (69).

In summary, we have verified our implementation of phonon and electron–phonon interaction by comparing results computed with Liouville’s equation and those obtained with DFPT, FPH, and PIMD methods. At the LDA/PBE level of theories, we obtain exactly the same results as with DFPT, as expected; at the hybrid functional level of theory, the results obtained with Liouville’s equation are comparable with those of the FPH and PIMD methods, with reasonable differences compatible with the different approximations employed in the three different approaches.

## 4 Electron–Phonon Renormalization of Energy Gaps in Small Molecules

**Figure 1:**
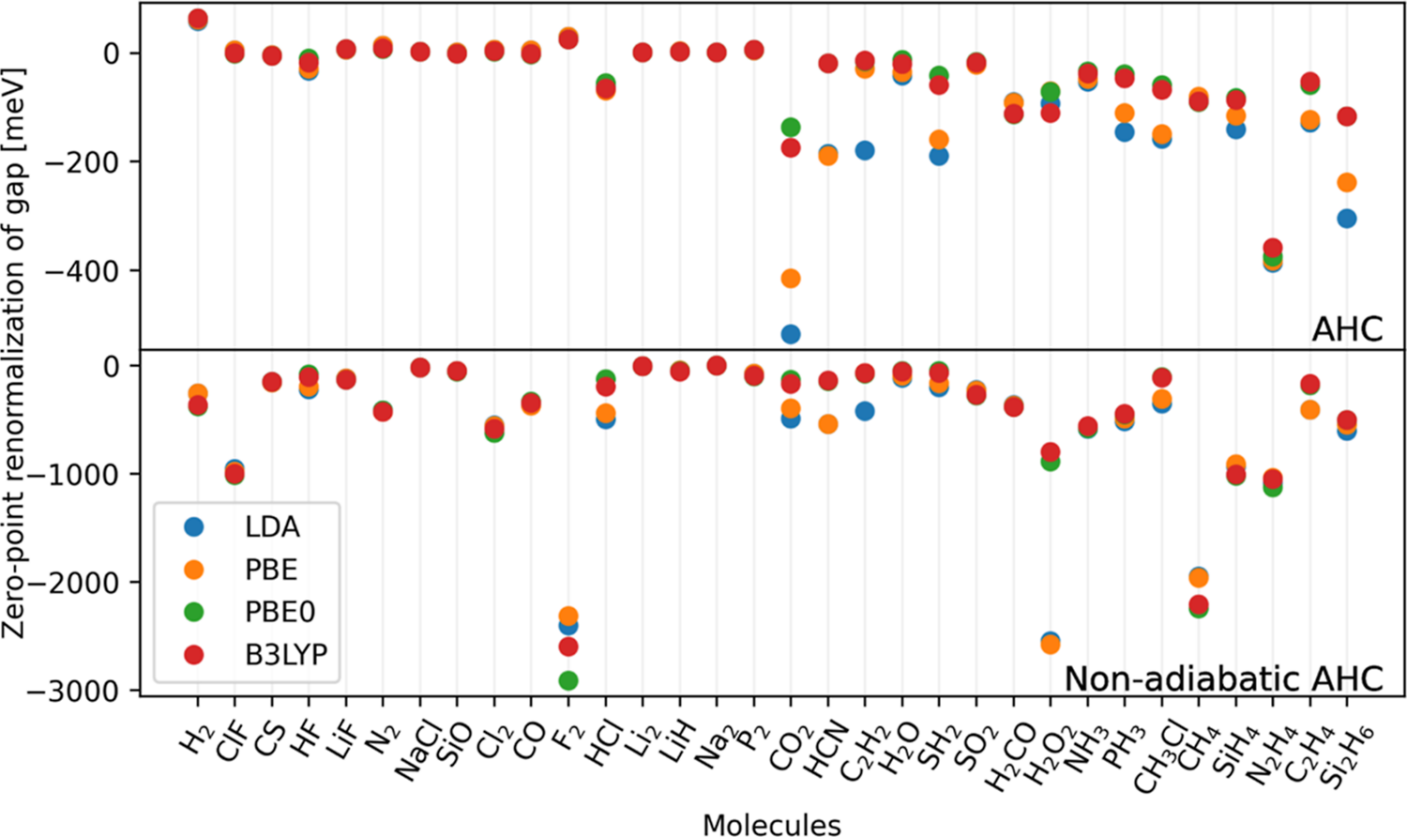
Computed ZPRs of the HOMO–LUMO gaps of small molecules using the AHC (upper panel) and NA-AHC (lower panel) approximations (see Table 1 for the definition of the approximations). We used different functionals specified in the inset.

Having verified our implementation, we carried out a study of the renormalization of the HOMO–LUMO gap of molecules in the G2/97 test set^71^ with LDA, PBE, PBE0, and B3LYP functionals, 50 Ry plane wave energy cutoff, and SG15^62^ ONCV^63^ pseudopotentials. The results are summarized in Tables 6 and 7 and are illustrated in Figure 1.

**Table 6:** HOMO–LUMO Energy Gaps of Small Molecules and Their Zero-Point Renormalization Energy (ZPR) Computed Within the Adiabatic AHC (A-AHC) approximation^a^

	LDA			PBE		PBE0		B3LYP		
Molecule	Gap	ZPR	Ref (69)	Gap	ZPR	Gap	ZPR	Gap	ZPR	Ref (69)
H_2_	9.998	0.058	–0.0021	10.164	0.061	11.890	0.063	11.648	0.063	0.0036
			0.0579^b^							
LiF	5.108	0.006	0.0331	4.723	0.006	7.014	0.007	6.601	0.007	0.0040
			0.0796^b^							
N_2_	8.221	0.013	0.0118	8.319	0.013	11.707	0.007	11.179	0.008	0.0087
			0.0130^b^							
CO	6.956	0.005	0.0065	7.074	0.004	10.055	–0.003	9.575	–0.002	0.0024
			0.0055^b^							
ClF	3.194	0.004	0.0041	3.167	0.005	6.250	–0.002	5.629	–0.001	0.0025
CS	3.954	–0.004	–0.0042	4.042	–0.004	6.562	–0.006	6.199	–0.006	–0.0058
HF	8.681	–0.032	–0.0397	8.598	–0.030	11.302	–0.011	10.809	–0.018	–0.0299
NaCl	3.524	0.002	0.0001	3.225	0.002	5.069	0.002	4.577	0.002	0.0004
SiO	4.524	0.001	–0.0019	4.549	0.001	6.764	–0.002	6.368	–0.002	–0.0032
Cl_2_	2.899	0.006	0.0063	2.894	0.006	5.503	0.002	4.887	0.003	0.0060
F_2_	3.495	0.030	0.0369	3.370	0.029	7.840	0.025	6.917	0.025	0.0329
Li_2_	1.532	0.001	0.0006	1.524	0.001	2.582	0.001	2.343	0.001	0.0007
LiH	2.985	0.002	–0.0066	2.873	0.003	4.424	0.001	4.117	0.002	–0.0061
Na_2_	1.564	0.001	0.0002	1.521	0.001	2.495	0.000	2.264	0.001	0.0000
P_2_	3.649	0.005	0.0021	3.644	0.005	5.537	0.005	5.107	0.005	0.0037
CO_2_	8.075	–0.517	–0.6807	8.033	–0.415	10.159	–0.137	9.708	–0.174	–4.0916
HCN	7.878	–0.185	–0.1412	7.930	–0.190	10.186	–0.020	9.806	–0.019	–0.1714
H_2_O	6.272	–0.042	–0.0806	6.208	–0.036	8.511	–0.013	8.084	–0.020	–0.0524
SH_2_	5.212	–0.189	–0.0360	5.238	–0.160	6.942	–0.042	6.593	–0.059	–0.2117
SO_2_	3.457	–0.019	–0.0178	3.414	–0.021	6.087	–0.016	5.596	–0.018	–0.0186
H_2_CO	3.470	–0.091	–0.0876	3.589	–0.092	6.451	–0.114	5.993	–0.111	–0.1005
H_2_O_2_	5.028	–0.093	–0.1290	4.887	–0.071	7.780	–0.072	7.505	–0.110	–0.2254
NH_3_	5.395	–0.053	–0.0611	5.304	–0.048	7.205	–0.035	6.825	–0.038	–0.0333
PH_3_	5.999	–0.146	–0.0592	5.946	–0.110	7.388	–0.039	7.056	–0.047	–0.2017
C_2_H_2_	6.703	–0.179	–0.1901	6.712	–0.029	8.181	–0.016	7.835	–0.014	–0.2327
CH_3_Cl	6.232	–0.158	–0.1441	6.210	–0.149	8.042	–0.059	7.691	–0.068	–0.1141
CH_4_	8.799	–0.084	–0.1147	8.820	–0.081	10.647	–0.091	10.320	–0.090	–0.0947
SiH_4_	7.727	–0.141	–0.6149	7.772	–0.115	9.440	–0.083	9.187	–0.086	–0.2027
N_2_H_4_	4.892	–0.386	–0.1169	4.866	–0.383	6.736	–0.375	6.426	–0.359	–0.0793
C_2_H_4_	5.654	–0.129	–0.1358	5.673	–0.123	7.592	–0.059	7.224	–0.053	–0.1194
Si_2_H_6_	6.364	–0.305	–0.5880	6.386	–0.238	7.874	–0.117	7.609	–0.117	–1.8723

a All gaps and ZPRs are in eV. We compare results obtained with different energy functionals (LDA, PBE, PBE0, and B3LYP) and we also report ZPRs from ref (69) and, in few cases, ref (60).

b Reference (60).

**Table 7:** HOMO–LUMO Gaps of Small Molecules and Their ZPR Computed Within the Non-Adiabatic AHC (NA-AHC) Approximation^a^

	LDA		PBE		PBE0		B3LYP	
Molecule	Gap	ZPR	Gap	ZPR	Gap	ZPR	Gap	ZPR
H_2_	9.998	–0.260	10.164	–0.263	11.890	–0.377	11.648	–0.366
LiF	5.108	–0.123	4.723	–0.122	7.014	–0.134	6.601	–0.134
N_2_	8.221	–0.418	8.319	–0.432	11.707	–0.418	11.179	–0.428
CO	6.956	–0.361	7.074	–0.373	10.055	–0.338	9.575	–0.346
ClF	3.194	–0.959	3.167	–0.985	6.250	–1.011	5.629	–1.000
CS	3.954	–0.151	4.042	–0.156	6.562	–0.155	6.199	–0.154
HF	8.681	–0.225	8.598	–0.194	11.302	–0.083	10.809	–0.111
NaCl	3.524	–0.021	3.225	–0.022	5.069	–0.022	4.577	–0.022
SiO	4.524	–0.052	4.549	–0.054	6.764	–0.056	6.368	–0.055
Cl_2_	2.899	–0.557	2.894	–0.560	5.503	–0.622	4.887	–0.589
F_2_	3.495	–2.405	3.370	–2.317	7.840	–2.914	6.917	–2.600
HCl	6.768	–0.501	6.784	–0.440	8.858	–0.128	8.417	–0.195
Li_2_	1.532	–0.007	1.524	–0.008	2.582	–0.010	2.343	–0.010
LiH	2.985	–0.049	2.873	–0.045	4.424	–0.055	4.117	–0.058
Na_2_	1.564	–0.002	1.521	–0.002	2.495	–0.002	2.264	–0.002
P_2_	3.649	–0.077	3.644	–0.079	5.537	–0.100	5.107	–0.096
CO_2_	8.075	–0.495	8.033	–0.398	10.159	–0.136	9.708	–0.174
HCN	7.878	–0.543	7.930	–0.541	10.186	–0.147	9.806	–0.138
H_2_O	6.272	–0.114	6.208	–0.095	8.511	–0.050	8.084	–0.061
SH_2_	5.212	–0.203	5.238	–0.166	6.942	–0.050	6.593	–0.069
SO_2_	3.457	–0.231	3.414	–0.234	6.087	–0.281	5.596	–0.274
H_2_CO	3.470	–0.364	3.589	–0.376	6.451	–0.386	5.993	–0.382
H_2_O_2_	5.028	–2.549	4.887	–2.582	7.780	–0.891	7.505	–0.799
NH_3_	5.395	–0.590	5.304	–0.566	7.205	–0.578	6.825	–0.562
PH_3_	5.999	–0.516	5.946	–0.493	7.388	–0.453	7.056	–0.450
C_2_H_2_	6.703	–0.420	6.712	–0.074	8.181	–0.080	7.835	–0.073
CH_3_Cl	6.232	–0.351	6.210	–0.307	8.042	–0.112	7.691	–0.116
CH_4_	8.799	–1.950	8.820	–1.961	10.647	–2.245	10.320	–2.210
SiH_4_	7.727	–0.931	7.772	–0.916	9.440	–1.019	9.187	–1.007
N_2_H_4_	4.892	–1.082	4.866	–1.038	6.736	–1.129	6.426	–1.050
C_2_H_4_	5.654	–0.408	5.673	–0.411	7.592	–0.184	7.224	–0.173
Si_2_H_6_	6.364	–0.607	6.386	–0.551	7.874	–0.506	7.609	–0.507

a All gaps and ZPRs are in eV. We compare results obtained with different energy functionals (LDA, PBE, PBE0, and B3LYP).

Table 6 summarizes the renormalizations computed with the A-AHC formalism. For most of the molecules, using hybrid functionals does not significantly change the gap renormalization relative to LDA or PBE results. For example, the energy gap renormalizations of the H_2_ molecule computed with LDA, PBE, PBE0, and B3LYP functionals are 58, 61, 63, and 63 meV, respectively. However, hybrid functionals do reduce the magnitude of gap renormalization in several systems, and CO_2_ and CH_3_Cl are representative examples. In CO_2_, the renormalization is reduced from the −415 meV (PBE) to −137 meV (PBE0) level of theory; in CH_3_Cl, it is reduced from −149 meV (PBE) to −59 meV (PBE0).

We report in Table 7 our results within the non-adiabatic AHC (NA-AHC) framework. The removal of the adiabatic approximation significantly influences the computed magnitude of the gap renormalization in most of the molecules, with some exceptions, for example, CO_2_. For example, the H_2_ gap renormalization computed using PBE0 varies from 63 meV (AHC) to −377 meV (NA-AHC). The most significant differences are found for the F_2_ and H_2_O_2_ molecules, where the gap renormalizations computed at the PBE0 level of theory are 25 and −72 meV, respectively, within the AHC approach and −2914 and −891 meV, when using the non-adiabatic AHC method.

We emphasize that neither the AHC nor the non-adiabatic AHC formalism correctly describes the self-energies in the full energy range, and thus, we suggest that the frequency-dependent self-energies should always be computed.

## 5 Electron–Phonon Renormalization of the Energy Gap of Diamond

We computed the electron–phonon renormalization of the energy gap in diamond within the AHC formalism and by computing the NA-FF self-energies self-consistently (see Table 1 and eq 30). The calculations for diamond were carried out in a 3 × 3 × 3 supercell with 60 Ry plane wave energy cutoff and SG15^62^ ONCV^63^ pseudopotentials.

**Figure 2:**
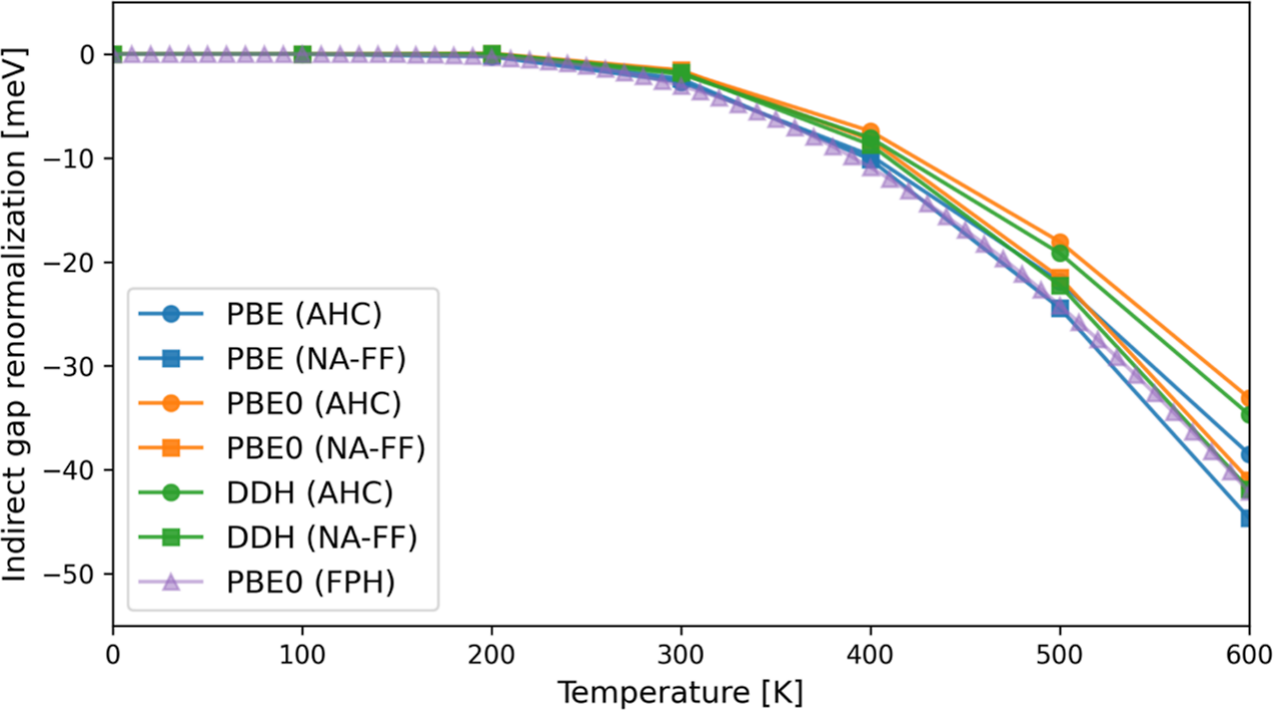
Electron–phonon renormalization energy of the indirect band gap of diamond computed by solving the Liouville equation and with different approximations to the self-energy, as defined in Table 1. The results obtained with the FPH approach and the PBE0 functional are also reported for comparison. The renormalization energy at zero temperature has been shifted to zero.

In Figure 2, we present the temperature-dependent indirect gap renormalization computed with the PBE, PBE0, and dielectric-dependent hybrid (DDH) functionals,^72,73^ where the fraction of exact exchange (0.18) in DDH is chosen to be the inverse of the dielectric constant of diamond (5.61).^72^ Within the same level of approximation, for example, the AHC formalism (circles in the plot), the PBE, PBE0, and DDH results are almost the same for temperatures lower than 400 K, but their difference increases at higher temperatures. With the same functional, for example, the PBE0 functional (orange lines in the plot), the results obtained with the fully frequency-dependent non-adiabatic self-energies are lower than those obtained with the AHC formalism. In general, the use of the hybrid functional does not significantly modify the trend of the ZPRs computed at the PBE level, as a function of temperature.

In Figure 2, we also report the renormalization of the indirect gap of diamond obtained with the FPH approach and the PBE0 functional. The results obtained with the FPH (purple line) approach and the Liouville equation (orange lines in the plot) are essentially the same below 300 K, but they differ as T is increased. The difference between the AHC/NA-FF and FPH approaches is always smaller than 10 meV at all temperatures, and it is reasonable considering that the FPH approach does not adopt the RIA, which is instead used within the AHC and NA-FF approaches.

**Figure 3:**
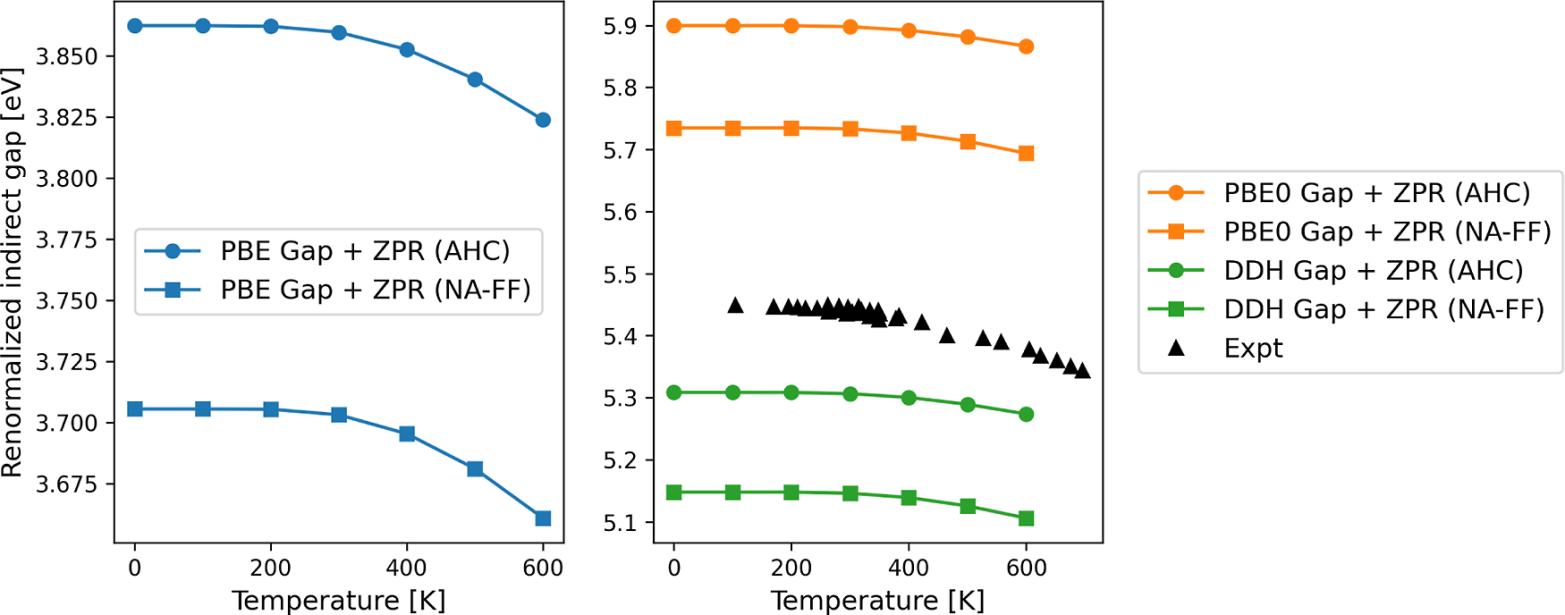
Electron–phonon renormalized indirect energy gap in diamond computed with the PBE and PBE0 functionals compared to experimental measurements.^74^ We show calculations performed with different approximations, as defined in Table 1.

A comparison of the computed and measured renormalized energy gap of diamond is given in Figure 3 and Table 8. Although the PBE0 and DDH hybrid functionals yield a similar trend as PBE for the electron–phonon renormalization as a function of temperature, the renormalized gap is noticeably improved compared to that of experiments when using hybrid functionals. The indirect energy gap of diamond computed with PBE, PBE0, and DDH without electron–phonon renormalization is 4.144, 6.189, and 5.597 eV, respectively, and the experimental indirect gap measured at approximately 100 K is 5.45 eV.^74^ By including electron–phonon renormalization, we can see that the results computed at the PBE0 level of theory agree relatively well with the experimental measurements (see Figure 3 and Table 8). The renormalized indirect gap computed with the PBE0 functional at 100 K is 5.899 eV when the AHC formalism is used, and it is 5.735 eV when the NA-FF self-energies are used. The renormalized indirect gaps computed with the DDH functional at 100 K are 5.308 (AHC) and 5.148 eV (NA-FF). As expected, the DDH results are closer to experimental measurements compared with those of the PBE0 functional since the fraction of exact exchange is chosen according to the system-specific dielectric constant. Overall, we find that computing electron–phonon interactions at the hybrid level of theory is a promising protocol to obtain quantitative results, comparable to those of experiments. We note that ref (19) reported −262 and −278 meV for the indirect gap renormalization of diamond obtained with the PBE functional, a 3 × 3 × 3 supercell, and the projector augmented-wave (PAW) method,^75^ using one-shot^76^ and standard^20,77^ Monte-Carlo (MC) approaches, respectively. These results are in good agreement with our result of −281 meV obtained with the AHC method at 0 K. Reference (19) also reported −320 and −315 meV with a 5 × 5 × 5 supercell, using one-shot and standard MC approaches, respectively, indicating that the full converged result is about 10% larger in absolute value relative to what obtained with a 3 × 3 × 3 supercell.

**Table 8:** Temperature-dependent ZPR and Renormalized Indirect Energy Gap (Gap + ZPR) Computed With the PBE, PBE0, and DDH Functionals, Using Different Levels of Approximations, as Defined in Table 1^a^

			Temperature [K]						
	Functional	Method	0	100	200	300	400	500	600
ZPR	PBE	AHC	–0.281	–0.281	–0.282	–0.284	–0.291	–0.303	–0.320
		NA-FF	–0.438	–0.438	–0.438	–0.441	–0.448	–0.463	–0.483
	PBE0	AHC	–0.290	–0.290	–0.290	–0.291	–0.297	–0.308	–0.323
		NA-FF	–0.454	–0.454	–0.454	–0.456	–0.463	–0.476	–0.495
	DDH	AHC	–0.289	–0.289	–0.289	–0.291	–0.297	–0.308	–0.324
		NA-FF	–0.450	–0.450	–0.450	–0.451	–0.458	–0.472	–0.492
Gap + ZPR	PBE	AHC	3.862	3.862	3.862	3.860	3.853	3.840	3.824
		NA-FF	3.705	3.705	3.705	3.703	3.695	3.681	3.661
	PBE0	AHC	5.899	5.899	5.899	5.898	5.892	5.881	5.866
		NA-FF	5.735	5.735	5.735	5.733	5.726	5.713	5.694
	DDH	AHC	5.308	5.308	5.308	5.306	5.300	5.289	5.274
		NA-FF	5.148	5.148	5.148	5.146	5.139	5.125	5.106

a The energy gaps computed at the PBE, PBE0, and DDH level of theory, without electron–phonon interaction, are 4.144, 6.189, and 5.597 eV, respectively. All energies are reported in eV.

## 6 Application to Spin Defects in Diamond

**Figure 4:**
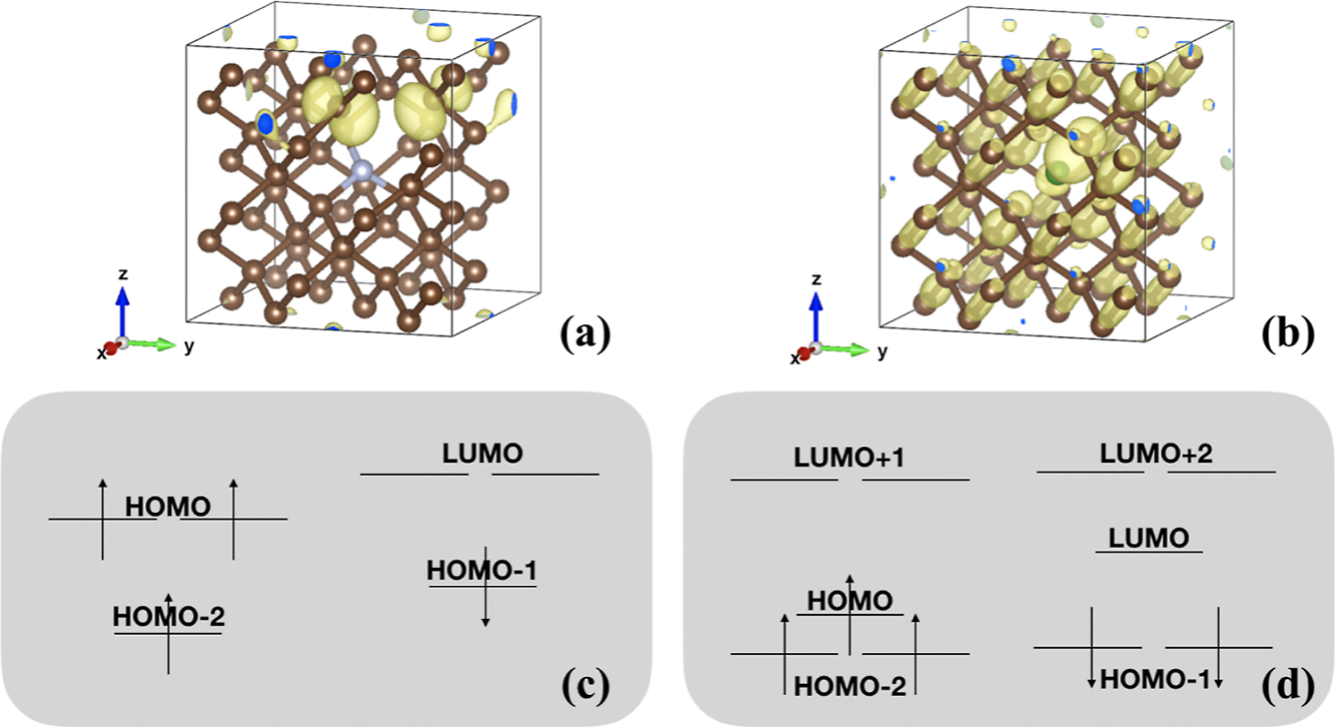
(a) Localized occupied state introduced by the nitrogen vacancy defect and (b) delocalized unoccupied state introduced by the single-boron vacancy defect. The wavefunctions are computed with the DDH functional. (c,d)Level ordering within the energy gap of diamond.

Spin defects have been extensively studied due to their potential applications in quantum technologies.^78−81^ To accurately predict the electronic structures of spin defects, we computed their electronic properties using electron–phonon renormalizations, and we considered a single-boron defect and the NV^–^ center shown in Figure 4. The calculations were carried out in a 2 × 2 × 2 cubic cell with a 60 Ry plane wave energy cutoff and SG15^62^ ONCV^63^ pseudopotentials (63 atoms for the NV^–^ center and 64 atoms for the single-boron defect).

In Tables 9 and 10, we report the electronic energy levels and ZPR for both defects obtained with the PBE, PBE0, and DDH functionals. We found that electron–phonon interactions weakly affect the energy levels of the NV^–^ center, which exhibit localized wavefunctions; they are instead more significant for the single-Boron defect with delocalized wavefunctions. In the NV^–^ center, the ZPR of the LUMO computed with the PBE functional is only −35 meV and that of the HOMO is negligible. In addition, the hybrid functionals PBE0 and DDH yield results similar to PBE results. For the boron defect, with the PBE (DDH) functional, the ZPRs of HOMO and LUMO are 111 meV (121) and 126 meV (241), respectively.

**Table 9:** Computed Energy Levels (eV) and Their ZPRs (eV) in the NV^–^ Center^a^

	PBE		PBE0		DDH	
	Level	ZPR	Level	ZPR	Level	ZPR
LUMO	1.359	–0.035	3.593	–0.033	2.948	–0.033
HOMO	0.000	0.001	0.000	0.012	0.000	0.009
HOMO – 1	–0.411	0.012	–0.059	0.004	–0.189	0.008
HOMO – 2	–0.924	0.038	–0.942	0.057	–0.952	0.052

a Energy levels are referred to the HOMO energy level, and the labels of energy levels are given in Figure 4c.

**Table 10:** Computed Energy Levels (eV) and Their ZPRs (eV) in the Boron Defect^a^

	PBE		PBE0		DDH	
	Level	ZPR	Level	ZPR	Level	ZPR
LUMO + 2	4.061	–0.359	6.109	–0.367	5.517	–0.365
LUMO + 1	4.041	–0.361	6.090	–0.368	5.498	–0.367
LUMO	0.137	0.126	1.389	0.285	1.027	0.241
HOMO	0.000	0.111	0.000	0.126	0.000	0.121
HOMO – 1	–0.278	0.087	–0.319	0.054	–0.308	0.062
HOMO – 2	–0.287	0.089	–0.327	0.104	–0.316	0.100

a Energy levels are referred to the HOMO energy level, and the labels of energy levels are given in Figure 4d.

## 7 Conclusions

In this paper, we computed phonon frequencies and electron–phonon interaction at the level of hybrid DFT by using DMPT and by solving the Liouville equation. Using this approach, we obtained phonon frequencies and energy gap renormalizations for molecules and solids by evaluating the non-adiabatic full frequency-dependent electron–phonon self-energies, thus circumventing the static and adiabatic approximations adopted in the AHC formalism, at no extra computational cost. We investigated the electronic properties of small molecules using LDA, PBE, B3LYP, and PBE0 functionals. We also carried out calculations of the electronic structure of diamond with the PBE, PBE0, and DDH functionals and found that the hybrid functionals PBE0/DDH noticeably improve the renormalized energy gap compared to experimental measurements. In addition, we studied the electron–phonon renormalizations of defects in diamond, and we concluded that electron–phonon effects are essential to fully understand the electronic structures of defects, especially those with relatively delocalized states. We note that the use of hybrid functionals affects both the perturbing potentials and wavefunctions entering the definition of the electron–phonon coupling matrices. In general, we expect the wavefunctions computed with hybrid functionals to be of better quality compared to those obtained with LDA/GGA. The perturbing potentials computed with hybrid functionals are non-local compared to the ones obtained with LDA/GGA. Thus, we expect both differences in wavefunctions and potentials, relative to LDA/GGA, to impact calculations with hybrid functionals.

In conclusion, computing electron–phonon interactions at the hybrid functional level of theory is a promising protocol to accurately describe the electronic structure of molecules and solids, and DMPT is a general technique that allows one to do so in an efficient and accurate manner by evaluating non adiabatic and full frequency-dependent electron–phonon self-energies.
